# Fractional-Order Identification of Gyroscope MEMS Noise Under Various Temperature Conditions

**DOI:** 10.3390/s24237504

**Published:** 2024-11-25

**Authors:** Dominik Sierociuk, Michal Macias, Konrad Andrzej Markowski

**Affiliations:** 1 Institute of Control and Industrial Electronics, Warsaw University of Technology, ul. Koszykowa 75, 00-662 Warsaw, Poland; dominik.sierociuk@pw.edu.pl (D.S.); konrad.markowski@pw.edu.pl (K.A.M.); 2 Military Electronic Works JSC, ul. 1 Maja 1, 05-220 Zielonka, Poland

**Keywords:** fractional calculus, fractional Kalman filter, estimation of fractional-order systems, fractional-order noise

## Abstract

This paper deals with identifying the fractional-order noise parameters for MEMS gyroscopes under various temperature conditions. The significant contribution of the paper is to investigate the relation between the fractional noise model of MEMS devices and different ambient temperatures. In our paper, variance, correlation, and introduced estimation analysis methods have been meticulously applied to determine noise parameters with fractional-order dynamics. Experimental data were collected precisely under various ambient temperatures, while the MEMS device was located in a climate chamber. The origin of the paper is motivated by a project entitled “Family of optoelectronic heads for guided missiles—SEEKER”, where the IMU sensor is a crucial electronic device used to measure the angular velocity of the optoelectronic head. It is widely known that the IMU measurements built-in MEMS technology often come with a random walk, as well as biases and noises affecting the final results.

## 1. Introduction

The accuracy improvement of measurements is a significant issue in real applications. The devices made using microelectromechanical system technology (MEMS), such as inertial measurement units, are undoubtedly related to noise affecting the actual value. Noise-decreasing attempts can effectively amend plenty of algorithms based on their measurements. However, complex dynamics can characterize such noise, and during so-far analysis, applying the methods based on fractional calculus has been found to successfully improve noise identification [1,2]. Therefore, this paper’s main contribution is to recognize and analyze how different ambient temperatures affect the gyroscope’s noise. So, a gyroscope’s noise readings were used to show the impact of the ambient temperature on fractional-constant and variable-order noise models, mainly investigating how the temperature conditions influence the order value occurring in such noise models.

**Figure 1:**
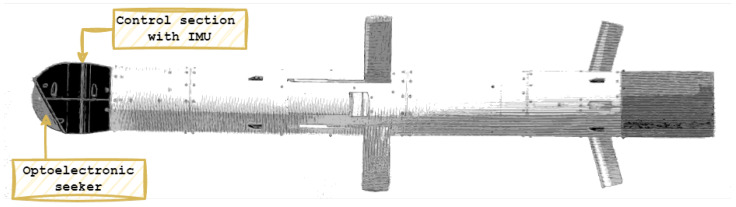
Composition of an anti-tank missile with the IMU placed in the front section.

In this paper, the deeply conducted and shown analysis of gyroscope MEMS noises was inspired by the project “Family of optoelectronic heads for guided missiles SEEKER” granted by the National Centre for Research and Development in Poland, where such a device was used to measure the angular velocity of the optoelectronic head. The sensor was situated to measure and control the angular velocities in both the azimuth and elevation directions. The optoelectronic head is designed for the missile to capture and track ground targets (stationary and moving) in day, night, and low-visibility conditions, considering the deterioration of operational parameters. One of many examples of this solution is the Spike missile [3]. The homing head of the missile is located in the nose section; behind it, there is an IMU unit with an electronics unit. The centre section contains the main engine and the main warhead, while the tail section contains the launch motor. Figure 1 shows the composition of a typical anti-tank missile with an optoelectronic seeker. In the project “Family of optoelectronic heads for guided missiles SEEKER”, the most important element used to orient the head in space includes the IMU unit (Inertial Measurment Unit), which is necessary for proper missile control. One of the important tasks in the work was the selection of the IMU sensor, which was modeled for tests using the HIL (Hardware In the Loop) method. As an exemplary sensor for the proof-of-concept procedure, the LSM6DSO32 IMU manufactured by STMicroelectronics. was used.

As shown in [4], IMU measurements are sensitive to thermal effects, which greatly influence their values. However, due to that effect, the previous paper achieved a complex mathematical model of IMU dynamics. Scientists try to apply various techniques to eradicate such an impact. An article [5] used, for example, an advanced type of recurrent neural network to model some parts of non-modeled MEMS gyroscope dynamics and apply this network into fractional-order sliding mode control. The paper [6] contains a survey of noise research in MEMS and explains its production mechanics. In the paper, the authors considered various types of MEMS sensors, e.g., optical MEMS, RF MEMS, chemical and biological MEMS, and others.

Fractional calculus is understood as a generalization of classical, integer-order differentiation and integration onto arbitrary-order operators. It is a theory of integrals and derivatives with real and even complex orders. The theoretical background can be found in [7,8,9,10]. Moreover, the Triple Estimation Algorithm (TEA) was introduced as a convenient tool for analysing, estimating, and identifying fractional-order systems. The main algorithm contains three fractional-order Kalman filters corresponding to each other. It is possible to achieve the fractional-order system parameters during algorithm application. In [1,2], the TEA algorithm was applied with success for the first time to MEMS noise identification coming from an accelerometer and gyroscope. Additionally, in [2], the TEA algorithm was extended to its finite length revision, improving the numerical efficiency and calculations.

The revealing of the unique properties of fractional calculus has caused engineers’ attention to this flexible and efficient tool. So, fractional calculus has been successfully adapted in the area of diffusion systems, where, for example, fractional-order models were used to characterize their behaviors [11,12]. Scientists have also discerned the potential of such mathematical branches in signal processing while designing new filters and analysis tools. Article [13] presents an adaptive filtering approach to filter the noise from MEMS measurements, where an adaptive Kalman filter was derived from the integer-order dynamic noise model. Some other applications based on fractional-order calculus in this area are presented in [10,14,15,16].

However, in some cases, applying fractional-order definitions containing time-varying orders is necessary to tackle systems with complex dynamics. There are so-called fractional variable-order definitions. Moreover, some fractional variable-order definitions can be presented based on switching schemes as their interpretations. A concise description of four switching schemes equivalent to four definitions of fractional variable-order derivatives is presented in [17,18]. The switching schemes representing order varying for selected variable order derivatives give insight into their behavior. The knowledge of order varying allows them to be categorized and conceived while implementing.

The paper is organized as follows. Its first part is devoted to basic fractional-order definitions and significant algorithms used in the identification process of noise order and its model. Section 2 introduces the Grünwald–Letnikov differ-integral definition and its approximation extended to different implementation lengths. The section also shows the discrete fractional-order state-space system and fractional-order Kalman filter. The recall of the fractional-order noise model and its identification methods are embraced in Section 3 and Section 4. Utilizing the variance, correlation, and estimation Kalman filter methods to identify the fractional-order noise, these methods are described in Section 4. Finally, in Section 5, the experimental setup and results from a meticulous investigation of an IMU denoted as LSM6DSO32 are used to identify the fractional-order noise parameters. The measurement data have been collected under various ambient temperatures adjusted in an environmental chamber. The precise temperatures used in the experiment cover the range between −40 °C and 60 °C. Then, based on the achieved modeling results, the figures show the model’s dependency on ambient temperature. The fractional-order noise model identification results are summarized in Section 6.

## 2. Fractional-Order Calculus

In this paper, the fractional constant-order Grünwald–Letnikov differ-integral definition is mainly used as a generalization of backward difference onto a non-integer order. Thus, the Grünwald–Letnikov difference can be formulated as follows [9,19]:

**Definition** **1.** *The fractional-order difference is given by the following equation:*

(1) Δαxk=∑j=0k(−1)jαjxk−j

*where α∈R is an order of the fractional difference, R is the set of real numbers, and k is a number of the sample for which the derivative is calculated. The factor αj is given by*

(2) αj=1for j=0α(α−1)…(α−j+1)j!for j>0

Definition 1 corresponds to a fractional constant-order derivative for α>0 or to a fractional constant-order integral for α<0. In a particular case, when α=0, it gives the original function.

For example, for a case of order α=0.5 and time sampling h=1, we obtain the following sum of all past samples:

$$\Delta^{0.5}x_{k}=1x_{k}-0.5x_{k-1}-0.125x_{k-2}-0.0625x_{k-3}\ldots$$

whereas for the first-order case, it is just the difference between two past samples.

This quite easily presents the main advantage of fractional-order differential operators compared to integer order operators; the fractional-order operators can describe better, more advanced dynamical relations with more complicated time relations.

During implementation processes, the number of samples taken into account is often constrained due to decreased computational complexity. Having the finite length of approximation *L*, it is possible to present the definition in the below finite length form:

(3) Δαxk=∑j=0L(k)(−1)jαjxk−j,

where,

(4) $$L\left(k\right)=\left\{\begin{array}{cc} k & \mathrm{if}\;k<L\\ L & \mathrm{if}\;k\geq L \end{array}\right.$$

The implementation length *L* impacts the accuracy of approximation of the fractional-order difference and also has an influence on the numerical complexity. The more significant number of samples taken into account during numerical implementation from one side improves the approximation accuracy. On the other side, it increases its computational complexity and is time-consuming. The value of approximation length should be selected carefully. However, there are no strict rules for its choosing. It depends on system dynamics and a usable time length that is taken into consideration; mostly, this parameter is chosen by experimental tests. Thus, it balances approximation accuracy and the algorithm’s execution regarding time consumption. In this paper, for comparison purposes, all models were run with two lengths: L=1000 and L=100.

### 2.1. Discrete Fractional-Order State-Space System (DFOSS)

It is possible to formulate a fractional constant-order state-space system based on the fractional constant-order definition. The DFOSS for various sets of equation order is described as follows:

**Definition** **2** **([20]).**  *The generalized discrete linear fractional order system with stochastic disturbances in a state-space representation is given by the following equations:*

(5) $$\begin{array}{ccc} \Delta^{\Upsilon }x_{k+1} & = & A_{d}x_{k}+Bu_{k}+\omega_{k}, \end{array}$$

(6) $$\begin{array}{ccc} x_{k+1} & = & \Delta^{\Upsilon }x_{k+1}-\sum_{j=1}^{k+1}{\left(-1\right)}^{j}\Upsilon_{j}x_{k+1-j}, \end{array}$$

(7) $$\begin{array}{ccc} y_{k} & = & Cx_{k}+\nu_{k}, \end{array}$$

*where*

Υk=diagα1k…αNk,

$$\Delta^{\Upsilon }x_{k+1}=\left[\begin{array}{c} \Delta^{\alpha_{1}}x_{1,k+1}\\ \vdots \\ \Delta^{\alpha_{N}}x_{N,k+1} \end{array}\right],$$

*where, $\nu_{k}$ and $\omega_{k}$ are independent noises with zero expected value; $\alpha_{1}\ldots \;\alpha_{N}$ are orders of system equations, and N denotes the number of these equations.*

### 2.2. Fractional Kalman Filter (FKF)

The estimation algorithm for fractional-order discrete state-space systems, being a generalization of the traditional Kalman Filter, has been meticulously introduced in [20] and is given as follows:

**Theorem** **1** **([20]).** *For the discrete fractional-order stochastic system in a state-space representation introduced by Definition 2, the simplified Kalman Filter (called the fractional Kalman filter) is given by the set of following equations:*

(8) $$\begin{array}{ccc} \Delta^{\Upsilon }{\tilde{x}}_{k+1} & = & A_{d}{\hat{x}}_{k}+Bu_{k} \end{array}$$

$$\begin{array}{ccc} {\tilde{x}}_{k+1} & = & \Delta^{\Upsilon }{\tilde{x}}_{k+1} \end{array}$$

(9) $$\begin{array}{ccc} & - & \sum_{j=1}^{k+1}{\left(-1\right)}^{j}\Upsilon_{j}{\hat{x}}_{k+1-j} \end{array}$$

$$\begin{array}{ccc} {\tilde{P}}_{k} & = & \left(A_{d}+\Upsilon_{1}\right)P_{k-1}{\left(A_{d}+\Upsilon_{1}\right)}^{T} \end{array}$$

(10) $$\begin{array}{ccc} & + & Q_{k-1}+\sum_{j=2}^{k}\Upsilon_{j}P_{k-j}\Upsilon_{j}^{T} \end{array}$$

(11) $$\begin{array}{ccc} {\hat{x}}_{k} & = & {\tilde{x}}_{k}+K_{k}\left(y_{k}-C{\tilde{x}}_{k}\right) \end{array}$$

(12) $$\begin{array}{ccc} P_{k} & = & \left(I-K_{k}C\right){\tilde{P}}_{k} \end{array}$$

*where*

$$K_{k}={\tilde{P}}_{k}C^{T}{\left(C{\tilde{P}}_{k}C^{T}+R_{k}\right)}^{-1}$$

*with initial conditions*

$$x_{0}\in R^{N},\;P_{0}=E\left[\left({\tilde{x}}_{0}-x_{0}\right){\left({\tilde{x}}_{0}-x_{0}\right)}^{T}\right]$$

*Moreover, the covariance matrix of an output noise $\nu_{k}$ in (7) and a system noise $\omega_{k}$ in (5) is defined, respectively, as*

(13) $$R_{k}=E\left[\nu_{k}\nu_{k}^{T}\right],\;Q_{k}=E\left[\omega_{k}\omega_{k}^{T}\right]$$

*and operator E defines the mathematical expected value.*

This algorithm was derived with the following assumptions:

$$E\left[x_{k+1-j}|z_{k}^{*}\right]\approx E\left[x_{k+1-j}|z_{k+1-j}^{*}\right]$$

fori=1,…,(k+1).

The expected values of terms $\left({\hat{x}}_{l}-x_{l}\right){\left({\hat{x}}_{m}-x_{m}\right)}^{T}$ are equal to zero when l≠m.

## 3. Fractional-Order Noise

Integer-order time-correlated (colored) noise is defined by the following relation:

(14) $$v_{k+1}=fv_{k}+\omega_{k}$$

where *f* denotes a noise parameter, $v_{k}$ is a time-correlated noise value, and $\omega_{k}$ is an uncorrelated noise value, for example, white Gaussian noise.

This can also be generalized for a fractional-order time correlation of noises. Colored fractional-order noise is defined as follows:

(15) $$\Delta^{\alpha }v_{k+1}=fv_{k}+\omega_{k}$$

where $v_{k}$ is a fractional colored noise value, α is an order of the noise, and $\omega_{k}$ is an uncorrelated noise.

For a fractional- or integer-order discrete system in state-space description with fractional-order colored noise, the fractional Kalman filter algorithm is given in this paper.

Equation (15) reveals interesting properties depending on the order and parameter values. When α=1, the equation becomes traditional integer-order color noise. In the case of f=0 and $\omega_{k}$ being white Gaussian noise, the given equation describes a realization of fractional Brownian motion and fractional Gaussian noise. Moreover, the constant-order α supplanted by time-varying values leads to a multifractional Brownian motion and multifractional Gaussian noise. The α value can be presented as a scalar or can be generalized to the vector form. Then, it is possible to achieve a particular order value for each single noise. Due to omitting the crosscorrelation between particular noises, the scalar *f* value in (15) can be supplanted by a diagonal *F* matrix.

## 4. Identification of Fractional Order Noise

Taking into account (15), we can rewrite it into the following matrix form:

(16) $$\left[\begin{array}{c} \Delta^{\alpha }v_{k+1}\\ \Delta^{\alpha }v_{k}\\ \vdots \\ \Delta^{\alpha }v_{1} \end{array}\right]=f\left[\begin{array}{c} v_{k}\\ v_{k-1}\\ \vdots \\ v_{0} \end{array}\right]$$

Assuming the predefined value of the order, we can solve that equation according to the parameter *f* using the Least Square method (LS).

(17) f=pinv(W(k))D(α,k+1)

where pinv is a pseudoinverse operation, and

$$W\left(k\right)=\left[\begin{array}{c} v_{k}\\ v_{k-1}\\ \vdots \\ v_{0} \end{array}\right]\;D\left(\alpha ,k+1\right)=\left[\begin{array}{c} \Delta^{\alpha }v_{k+1}\\ \Delta^{\alpha }v_{k}\\ \vdots \\ \Delta^{\alpha }v_{1} \end{array}\right]$$

The error of the solution of this equation is a source noise $\omega_{k}$ obtained for parameter *f* and order α:

(18) $$\omega_{k}^{*}=\Delta^{\alpha }v_{k+1}-fv_{k}$$

### 4.1. Mixed Noise Case

In practical cases, fractional-order noise can be mixed with other dynamically uncorrelated noises. In such a case, the noise equation has the following form:

(19) $$\begin{array}{ccc} \Delta^{\alpha }x_{k+1} & = & fx_{k}+\omega_{k} \end{array}$$

(20) $$\begin{array}{ccc} v_{k} & = & x_{k}+\nu_{k} \end{array}$$

In order to define the equation for the LS algorithm, let us apply the difference operator to the output equation:

(21) $$\Delta^{\alpha }v_{k+1}=\Delta^{\alpha }x_{k+1}+\Delta^{\alpha }\nu_{k+1}.$$

Substituting the system equation, we obtain

(22) $$\Delta^{\alpha }v_{k+1}=fx_{k}+\omega_{k}+\Delta^{\alpha }\nu_{k+1}$$

and then substitute $x_{k}$ using the output equation:

(23) $$\Delta^{\alpha }v_{k+1}=fv_{k}-f\nu_{k}+\omega_{k}+\Delta^{\alpha }\nu_{k+1},$$

This can be rewritten in the same way as the previous Least Square form:

(24) $$\left[\begin{array}{c} \Delta^{\alpha }v_{k+1}\\ \Delta^{\alpha }v_{k}\\ \vdots \\ \Delta^{\alpha }v_{1} \end{array}\right]=f\left[\begin{array}{c} v_{k}\\ v_{k-1}\\ \vdots \\ v_{0} \end{array}\right]$$

The difference is only in terms of the equation error.

(25) $$\omega_{k}^{*}=-f\nu_{k}+\omega_{k}+\Delta^{\alpha }\nu_{k+1}.$$

The error is not only the source noise but also the difference between the output noise and a linear part of that noise. This leads to problems with order identification, because it is hard to determine the minimization cost function, which allows the proper diagnosis of the noise order. To deal with it, we focused on variance and correlation methods.

The variance method analyses the value of the identification equation error variance, assuming that when the variance is minimal, the model performs best because it transforms the low variance noise into the required noise.

The correlation method is based on analyzing the time-correlation coefficients of the equation error (source noise), with the assumption that the best performance of the model will be achieved at the minimal values of time correlation, as was presented in [21]. The objective function is defined as a sum of the normalized correlation coefficient given by

(26) $$J=\sum_{m=0}^{k}\left|R_{\omega^{*}}\left(m\right)\right|,$$

where

(27) $$R_{\omega^{*}}\left(m\right)=E\left\{\omega_{k+m}^{*}\omega_{k}^{*}\right\}=E\left\{\omega_{k}^{*}\omega_{k-m}^{*}\right\},$$

and *m* is the shift between samples.

Both methods can be used to determine the noise order by numerical minimization of the objective functions.

### 4.2. Estimation Analysis—Alternative Method

The fractional mixed noise model given by the (19) and (20) matrices adapted to the fractional Kalman filter are as follows:

(28) $$\begin{array}{ccc} A & = & \left[f\right],\;B=\left[0\right] \end{array}$$

(29) $$\begin{array}{ccc} C & = & \left[1\right],\;N=\left[\alpha \right] \end{array}$$

The parameter matrices are given as $Q=[var\left(\omega^{*}\right)]$, R=γQ, where γ is a relation coefficient between matrices *R* and *Q*. The *R* to *Q* coefficient allows for the modeling of different relations between *R* and *Q*, which allows for testing scenarios when the filter takes into account the prediction based on the model with higher or lower probability, according to the Kalman Filter cost function:

$$\begin{array}{ccc} {\hat{x}}_{k} & = & \arg\min_{x}[\left({\tilde{x}}_{k}-x\right){\tilde{P}}_{k}^{-1}{\left({\tilde{x}}_{k}-x\right)}^{T} \end{array}$$

(30) $$\begin{array}{ccc} & + & \left(y_{k}-Cx\right)R_{k}^{-1}{\left(y_{k}-Cx\right)}^{T}] \end{array}$$

The main problem solved by this method is to determine the most accurate model (order of the model) of the noise. The higher the value of the *R* to *Q* coefficient, the more important role in estimation the model will play. This will be used to observe the fractional Kalman filter efficiency for such a value of fractional order, which can be interpreted as an efficiency of the model for such an order, and finally, to determine the fractional order of the noise.

The error is defined as a difference between the estimated and measured output of the system.

(31) $$e_{k}={\hat{x}}_{k}-y_{k}$$

The more adequate the noise model, the less error for higher *R* values that will be achieved.

## 5. Experimental Results

As mentioned in the introduction, the main idea of this paper is to analyze how parameters of the noise change during various temperature conditions. This is especially important for observing if the fractional order of the noise varies with the temperature, which implies the necessity of using variable-order calculus, which is much more complex than in a constant-order case, or when the order changing can be omitted, the constant fractional-order model can be used.

To measure the noise without additional filtering or post-processing, it was necessary to obtain the native sampling time of the LSM6DSO32 MEMS sensor, which is equal to 6660 Hz. Such a high value of sampling time implies the requirement for a very high transfer rate between the sensor and the data-collecting board. In the experiment, the board Arduino Portenta with Arduino Portenta Breakout was used to connect the sensor through the SPI interface, temporarily transfer data to RAM, and save data on an SD Card.

The conditions in the environmental chamber were set to 0% humidity and an atmosphere pressure of 1012 hPa. Such experiment conditions let us avoid the rime on the device surface during the test in minus temperatures and prevent it from damage.

According to the standard [22] for testing components, like IMU sensors used as subassemblies in missiles, climate tests in the chamber are conducted for operation at an elevated ambient temperature of +55 °C and a reduced ambient temperature of −40 °C.

### 5.1. Experimental Setup

**Figure 2:**
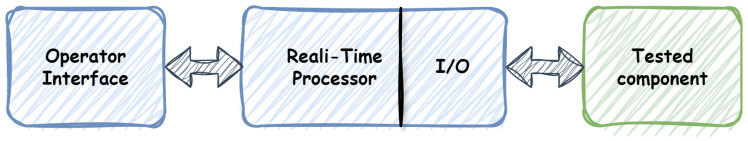
An HIL test system.

In engineering work, three testing methods are most commonly used: MIL (Model In the Loop), SIL (Software In the Loop) and HIL (Hardware In the Loop). This work used the HIL research method to test the IMU sensor. A HIL test system consists of three primary components: a real-time processor, I/O interfaces, and an operator interface (Figure 2). The real-time processor is the core of the HIL test system. It provides system components such as hardware I/O communication, data logging, etc. The I/O interfaces are analog, digital, and bus signals interacting with the unit under test. The operator interface communicates with the real-time processor to provide test commands and visualization.

**Figure 3:**
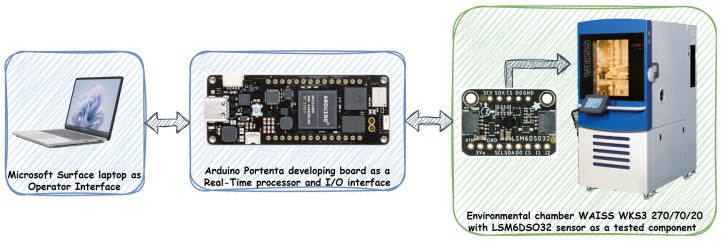
Environment setup.

In our case, the environment setup consisted of the following parts (Figure 3): Microsoft Surface laptop as a Operator Interface; Arduino Portenta developing board with dual-core STMicroelectronics STM32H747 processor as a Real-Time processor and I/O interfaces; and an environmental chamber Weiss Technik Company type WAISS WKS3 270/70/20 with STMicroelectronics LSM6DSO32 sensor as a tested component. All elements of the environmental configuration are presented in detail below.

#### 5.1.1. LSM6DSO32 MEMS Sensor

The LSM6DSO32 sensor discerning in the experimental setup is an inertial measurement unit (IMU) made using microelectromechanical (MEMS) technology. The investigated IMU sensor contains three accelerometers and three gyroscope axes with a maximum sampling frequency of 6.6 kHz. Hobbyists widely use the LSM6DSO32 IMU sensor to measure the linear velocity and angular rate. The range of three accelerometer axes can be adjusted within selected sets, up to ±32 g, and the range of three gyroscope axes can be adjusted up to ±2000 dps. The unit possesses two convenient communication interfaces: I2C and SPI. Additionally, the data originating from gyroscope and accelerometer axes can be used in such algorithms as Attitude and Heading Reference Systems (AHRSs) and Inertial Navigation Algorithms (INSs), giving the objects’ location and orientation.

#### 5.1.2. Arduino Portenta

The main parts of the Arduino Portenta developing board used in the experimental setup and during data collection contain a dual-core STM32H747 processor with a graphics engine and 8 MB SDRAM. The developing board also includes three A/D Converters with 16-bit resolution and two 12-bit D/A converter. Due to its plenty of miscellaneous, it is a capable and efficient programming unit.

#### 5.1.3. Environmental Chamber

An environmental test chamber, also called a climatic chamber or climate chamber, artificially replicates conditions to which components such as electronics might be exposed. Chamber testing involves validation and exposing products such as electronics components to various environmental conditions in a laboratory-controlled setting. An electronic component is placed inside the chamber and subjected to one or more of these environmental parameters to measure the operational reliability of a testing component. The WAISS climatic chamber type WKS3 270/70/20 was used to test the LSM6DSO32 MEMS sensor. The performance data of the climatic chamber used in the experiment are presented in Table 1.

### 5.2. Data Collection

Data for one axis (x axis) of the gyrosensor were collected together with temperature data for the following temperatures: −40 °C,−30 °C, −20 °C, −10 °C, +10 °C, +20 °C, +40 °C, and +60 °C. In order to collect data only for the sensor’s noise, the chamber was turned off during the data collection process. The data was collected in a static situation to analyze the native sensor noise (for example, without the influence of acceleration on gyro measurement).

**Figure 4:**
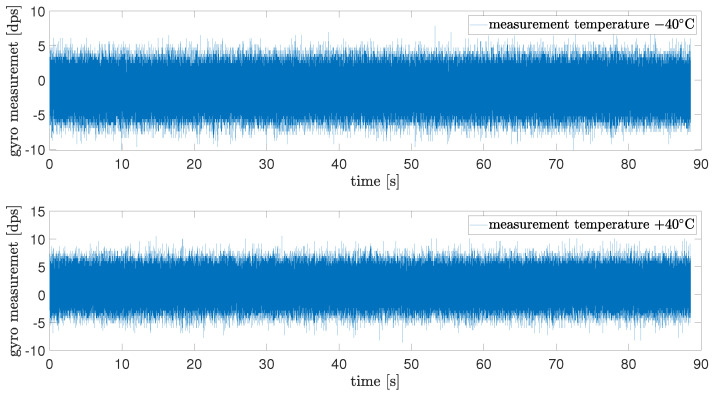
Examplary measurements for two different temperatures.

Two examples of collected data for different temperatures (−40 °C and +40 °C) are presented in Figure 4. As it can be noticed, both measurements differ in offset and variance. This paper considered the modeling of the measurement noise, which is why acquired data from the gyrosensor were post-processed to extract only the noise by subtracting offset. The difference in the variance of measured noise between two edge cases −40 °C and +60 °C equaled 18%.

### 5.3. Variance and Correlation Results

**Figure 5:**
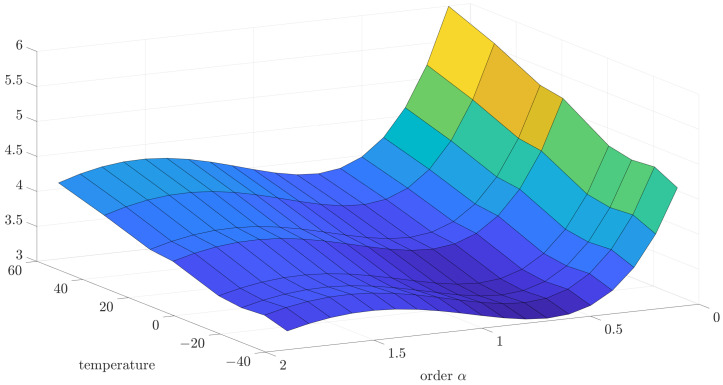
The 3D visualization of the identified source noise variance for different values of order and implementation length L=1000.

**Figure 6:**
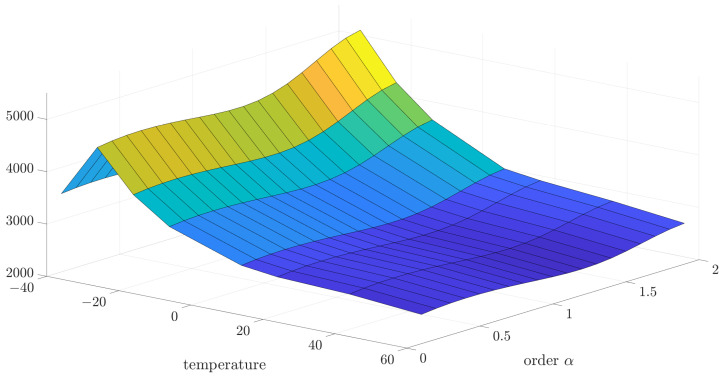
The 3D visualization of the identified source noise correlation coefficient for different values of order and implementation length L=1000.

Initially, the collected data were analyzed using the variance and correlation method. In this case, the implementation length of *L* was equal to 1000. For a better presentation of a general trend in the temperature dependency of variance and correlation, 3D mesh plots are presented in Figure 5 and Figure 6. However, for a better presentation of the minimum function, which will be more useful to determine the main result—the noise order—the 2D plots have been used.

**Figure 7:**
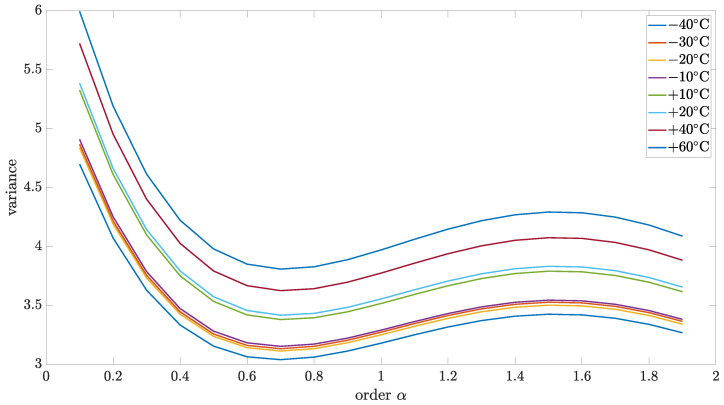
Variance of identified source noise for different values of order and implementation length L=1000.

**Figure 8:**
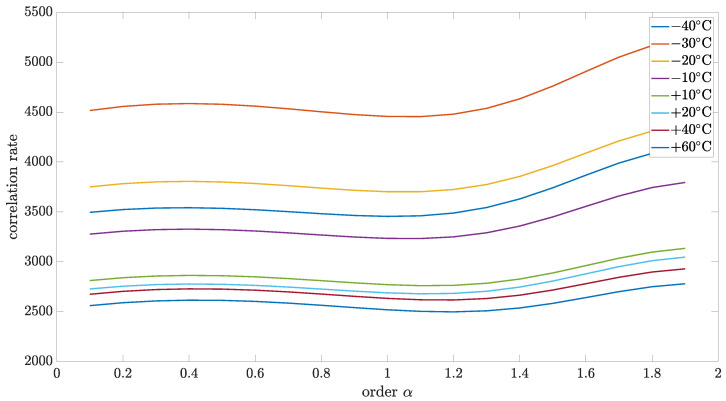
Correlation coefficient of identified source noise for different values of order and implementation length L=1000.

**Figure 9:**
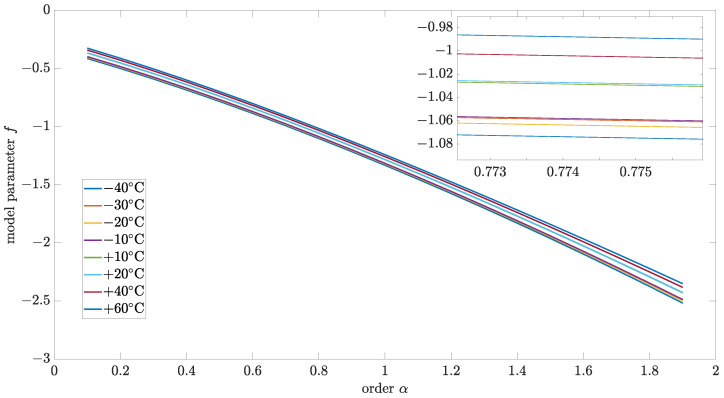
Parameters of identified source noise for different values of order and implementation length L=1000.

Figure 7 and Figure 8 show the results of variance and correlation methods, respectively. The identified parameters of source noise for different order values were presented in Figure 9. Additionally, the results of the variance and correlation methods for L=1000 are summarized in Table 2 and Table 3. As it is worth noticing, the presented results show no significant difference between the obtained results for different temperatures.

#### Results for Shorter Implementation Length L=100

The implementation length can influence the numerical results of fractional order differences; that is why it would be interesting to check if taking a shorter implementation length would change the results in comparison with those obtained for L=1000. The results of the variance and correlation methods for L=100 are summarized in Table 4 and Table 5. As can be noticed, there are no differences in the order and marginal differences in the minimum value for all temperatures. This implies the conclusion that the model obtained in this way can be less numerically demanding.

### 5.4. Estimation Test Results

**Figure 10:**
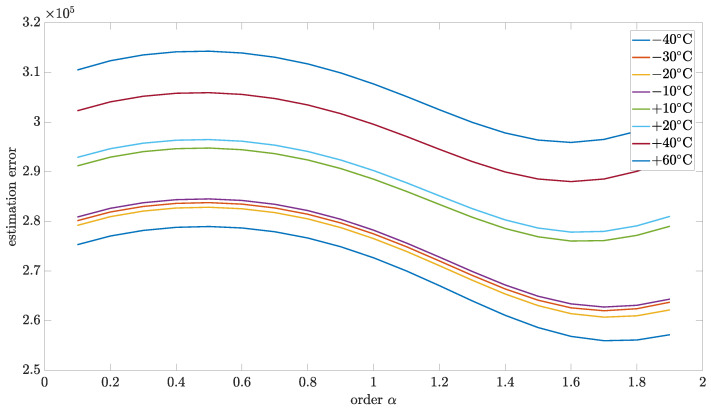
Estimation test results for R to Q coefficient equal to 0.5 presented for different temperatures.

**Figure 11:**
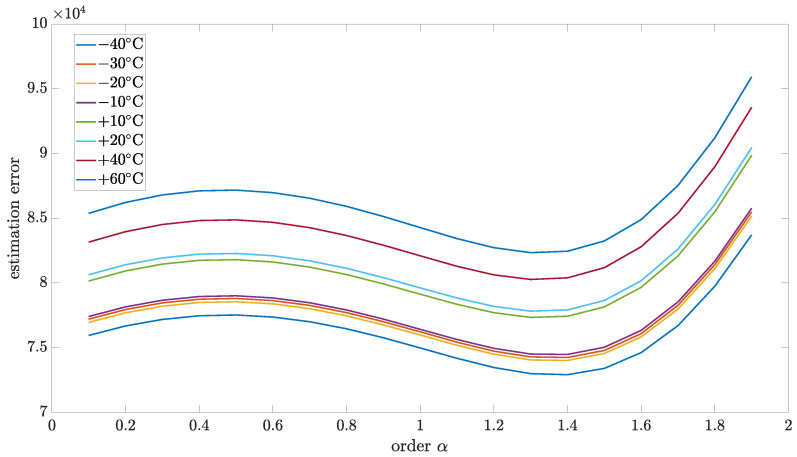
Estimation test results for R to Q coefficient equal to 0.1 presented for different temperatures.

**Figure 12:**
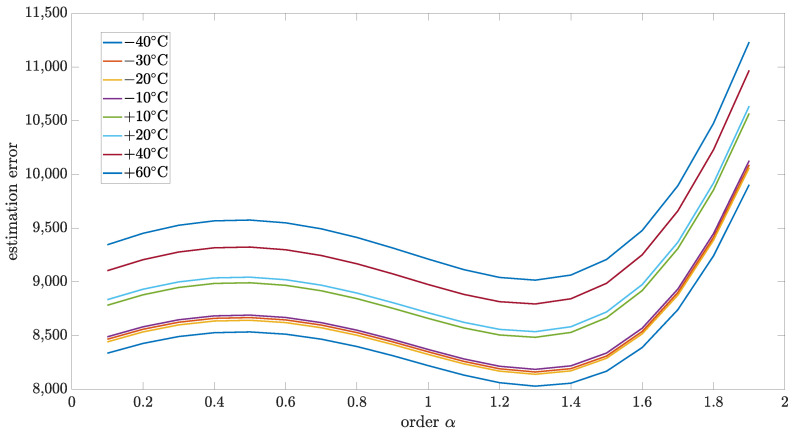
Estimation test results for R to Q coefficient equal to 0.01 presented for different temperatures.

**Figure 13:**
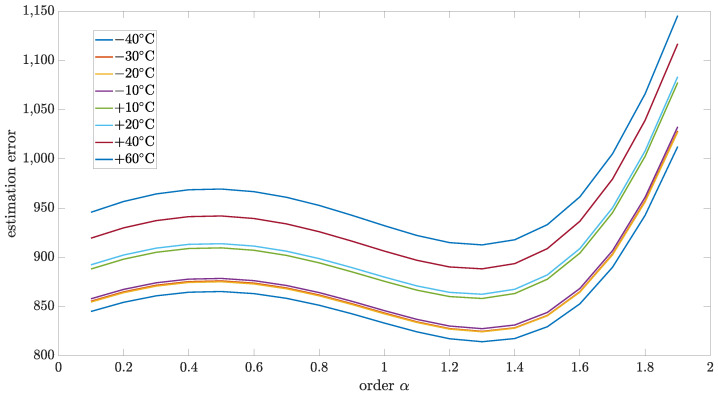
Estimation test results for R to Q coefficient equal to 0.001 presented for different temperatures.

This section presents the results of the estimation method, which is proposed in this paper. The relationship between matrix *R* and *Q* was used to analyze the model robustness for the particular order. Figure 10, Figure 11, Figure 12 and Figure 13 present estimation results for the relations between matrix *R* and *Q* equal to 0.5, 0.1, 0.01, and 0.001, respectively. Additionally, Table 6, Table 7, Table 8 and Table 9 encompass the estimation errors under various tested temperatures and model orders. With the lower value of this coefficient, the Kalman filter is more optimized for data obtained in model-based prediction. The minimum estimation error will be interpreted as the most accurate mode (order of the model). Also, the important observation that will be able to make is how the identified order will change when the temperature changes. All of those results were obtained for implementation length L=100.

**Figure 14:**
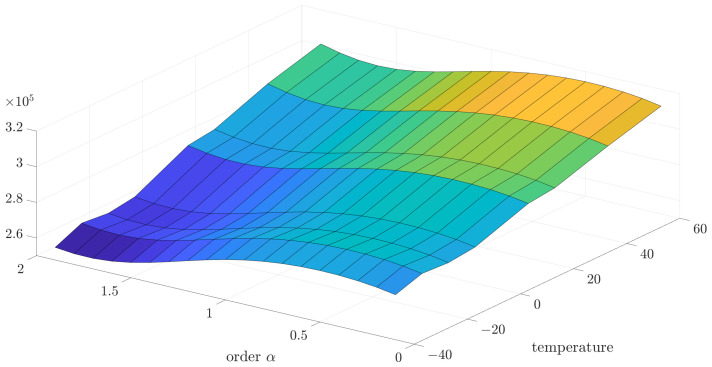
The 3D visualization of the estimation test results for R to Q coefficient equal to 0.5 presented for different temperatures.

Figure 14 presents the 3D plot of estimation test results to better present the general dependency of estimation error to order and temperature. Otherwise, for better exposition of minimum estimation results, the 2D plots have been presented for this and other cases of the relationship between matrix *R* and *Q*.

As can be seen, for all cases, the identified order did not significantly change with temperature changes. From the highest to the lowest temperature, the identified order changes were in the range of 0.1, which was the step of the tested set.

For the R to Q relation equal to 0.5, the identified order was in a different range than for other cases. Moreover, it was shifted in the direction of the second order, which can lead to an unstable model which is not physical. Probably, for cases with a higher value of R to Q relation, a more complex model with more than one state variable will be required (commensurate or non-commensurate orders). For R to Q relation equal 0.1, the identified order had values 1.3 and 1.4, but the cumulative result for all tested temperatures, as presented in Table 10, was equal to 1.3. For cases of the R to Q relation equal to 0.01 and 0.001, the, the identified order equaled 1.3 for all tested temperatures.

## 6. Conclusions

The paper presents the experimental results of fractional-order noise parameter identification for different environment temperatures. The main purpose of the presented analyses was to recognize if the noise order would vary with temperature. During the identification process, three methods were applied to achieve the parameters of the fractional-order noise model. The noise from the MEMS sensors’ measurement creates a complex problem during identification and analysis. Due to this, we used the variance, correlation, and estimation methods based on fractional-order Kalman filter methods to describe the noise dynamic under various ambient temperatures. Moreover, the experimental results for various implementation lengths of fractional differences occurring in the noise model were presented for all applied methods. Combining all experiments together, the general conclusion emerges that the fractional order of the gyroscope noise model determined from the LSM6DSO32 device is not so much affected by the ambient temperature while it is stationary. The analysis and research were conducted under various temperatures in the environmental chamber. The comparison between different models, such as integer-, non-integer- constant-, and variable-order, was also made to provide thorough insight into experimental and fractional order modelling results. Comparison results for the integer-, fractional- and variable fractional-order models are presented in Table 11.

The obtained results show that the increasing effectiveness between integer and fractional constant order is 2.4054%; however, between constant fractional and variable fractional order is only 0.031841%. In such a case, the effectiveness of the fractional constant-order model is very similar to the variable-order one. It can be helpful knowledge from a computational complexity point of view in future applications.

**Figure 15:**
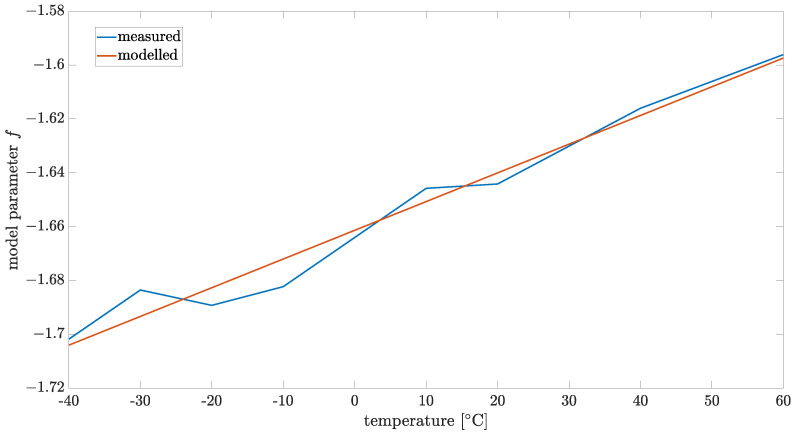
Modeled parameter for order α=1.3.

Finally, the obtained model is a constant-order non-stationary described by the following form:

(32) $$\Delta^{1.3}v_{k+1}=f\left(T\right)v_{k}+\omega_{k},$$

where f(T)=0.0011T−1.6614 is a noise parameter for temperature *T*. Parameters of the function f(T) were identified from measured data by the LS method and are presented in Figure 15.

**Figure 16:**
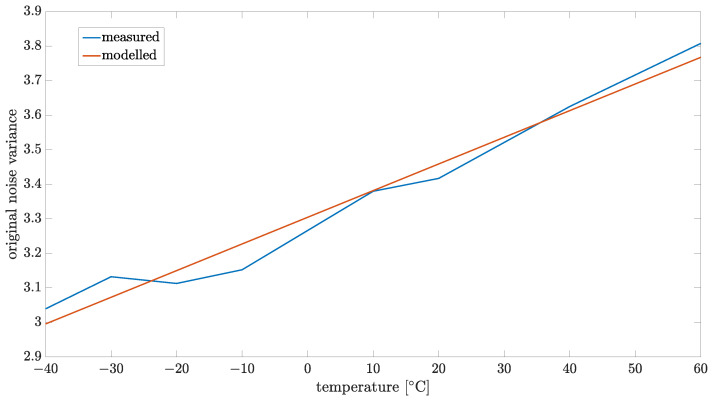
Modeled variance of original noise $\omega_{k}$ for order α=1.3.

The variance in the original noise $\omega_{k}$ also depends on temperature with the following relation: $var(\omega_{k})=0.0077T+3.3045$; this was identified from measured data using the LS method. A comparison of the measured and modeled function is presented in Figure 16.

Moreover, the obtained results of the identified order for the correlation method are much closer to those obtained by the estimation method than for the variance method. This can suggest that for coarse determination of the order in future research, the correlation method can be used, and then for final order determination, the estimation method has to be used. For more accurate results, the Triple Estimation method for identifying together system order, parameters, and state variables can be used in future research with initial values of parameters and orders obtained during the estimation method.

Obtained in such a way, the noise model can be used for HIL simulations and to construct more efficient estimation algorithms (e.g., AHRS algorithms).

## Figures and Tables

**Table 1:** The performance data of the WKS3 270/70/20.

Test Space Dimension H×W×D	Minimum Temperature	Maximum Temperature	Temperature-Changing Rate Cooling	Temperature-Changing Rate Heating	Temperature Deviation in Time
mm	°C	°C	K/min	K/min	K
750×580×615	−72	+180	20.0	20.0	±0.1 to ±0.5

**Table 2:** Results for variance method and L=1000.

Temp.	−40 °C	−30 °C	−20 °C	−10 °C	+10 °C	+20 °C	+40 °C	+60 °C
**Variance**	3.0394	3.1324	3.1129	3.1526	3.3799	3.4171	3.6252	3.8079
**Order**	0.7	0.7	0.7	0.7	0.7	0.7	0.7	0.7

**Table 3:** Results for correlation method and L=1000.

Temp.	−40 °C	−30 °C	−20 °C	−10 °C	+10 °C	+20 °C	+40 °C	+60 °C
**Corr. coeff.**	3454.9	4455.2	3702.1	3232.8	2760.5	2679	2617.1	2497.9
**Order**	1	1.1	1	1.1	1.1	1.1	1.2	1.2

**Table 4:** Results for variance method and L=100.

Temp.	−40 °C	−30 °C	−20 °C	−10 °C	+10 °C	+20 °C	+40 °C	+60 °C
**Variance**	3.0394	3.1324	3.1129	3.1526	3.3799	3.4171	3.6252	3.8080
**Order**	0.7	0.7	0.7	0.7	0.7	0.7	0.7	0.7

**Table 5:** Results for correlation method and L=100.

Temp.	−40 °C	−30 °C	−20 °C	−10 °C	+10 °C	+20 °C	+40 °C	+60 °C
**Corr. coeff.**	3454.9	4455.1	3702.1	3232.8	2760.5	2679	2617	2497.9
**Order**	1	1.1	1	1.1	1.1	1.1	1.2	1.2

**Table 6:** Results of estimation method for L=100 and *R* to *Q* relation equal to 0.5.

Temp.	−40 °C	−30 °C	−20 °C	−10 °C	+10 °C	+20 °C	+40 °C	+60 °C
**Est. error**	255,983	262,015	260,727	262,765	276,054	277,849	288,025	295,911
**Order**	1.7	1.7	1.7	1.7	1.6	1.6	1.6	1.6

**Table 7:** Results of estimation method for L=100 and *R* to *Q* relation equal to 0.1.

Temp.	−40 °C	−30 °C	−20 °C	−10 °C	+10 °C	+20 °C	+40 °C	+60 °C
**Est. error**	72,907	74,238	74,007	74,470	77,332	77,817	80,266	82,335
**Order**	1.4	1.4	1.4	1.4	1.3	1.3	1.3	1.3

**Table 8:** Results of estimation method for L=100 and *R* to *Q* relation equal to 0.01.

Temp.	−40 °C	−30 °C	−20 °C	−10 °C	+10 °C	+20 °C	+40 °C	+60 °C
**Est. error**	8029.4	8161.2	8139.8	8185.9	8484.2	8536.6	8794.5	9016.3
**Order**	1.3	1.3	1.3	1.3	1.3	1.3	1.3	1.3

**Table 9:** Results of estimation method for L=100 and *R* to *Q* relation equal to 0.001.

Temp.	−40 °C	−30 °C	−20 °C	−10 °C	+10 °C	+20 °C	+40 °C	+60 °C
**Est. error**	814.1	824.7	824.2	827.4	858.1	862.4	888.2	912.5
**Order**	1.3	1.3	1.3	1.3	1.3	1.3	1.3	1.3

**Table 10:** Estimation test errors.

R to Q Relation	Estimation Error	Order
0.001	$6.8116\times 10^{3}$	1.3000
0.01	$6.7348\times 10^{4}$	1.3000
0.1	$6.1357\times 10^{5}$	1.3000
0.5	$2.1807\times 10^{6}$	1.7000

**Table 11:** Comparison results for R to Q relation γ=0.1.

Model Type	Estimation Error
integer order	628,689.9070
constant fractional order	613,567.6493
variable fractional order	613,372.2834

## Data Availability

Data are contained within the article.
